# Supplementary material for: Single Cell Transcriptomic and Chromatin Profiles Suggest Layer Vb Is the Only Layer With Shared Excitatory Cell Types in the Medial and Lateral Entorhinal Cortex
Source: Front Neural Circuits. 2022 Jan 26;15:806154. doi: 10.3389/fncir.2021.806154 (PMC8826650; doi:10.3389/fncir.2021.806154)
Supplement: Supplementary file 14 [file Data_Sheet_1.ZIP › MEC_Sup_vs_LEC_Sup_Motifs/homerResults/motif20.similar.html]

motif20

## Information for motif20

T
C
A
G
G
C
A
T
C
G
T
A
C
A
T
G
T
A
C
G
T
G
A
C
G
T
A
C
G
C
T
A
A
T
G
C
C
A
T
G
A
T
G
C
G
A
T
C
  
Reverse Opposite:  

C
T
A
G
T
A
C
G
G
T
A
C
T
A
C
G
C
G
A
T
C
A
T
G
A
C
T
G
A
T
G
C
G
T
A
C
G
C
A
T
C
G
T
A
A
G
T
C
  

|  |  |
| --- | --- |
| p-value: | 1e-34 |
| log p-value: | -7.937e+01 |
| Information Content per bp: | 1.530 |
| Number of Target Sequences with motif | 46.0 |
| Percentage of Target Sequences with motif | 0.22% |
| Number of Background Sequences with motif | 2.8 |
| Percentage of Background Sequences with motif | 0.02% |
| Average Position of motif in Targets | 319.6 +/- 205.8bp |
| Average Position of motif in Background | 331.5 +/- 62.3bp |
| Strand Bias (log2 ratio + to - strand density) | -0.5 |
| Multiplicity (# of sites on avg that occur together) | 1.00 |
| Motif File: | file (matrix) reverse opposite |

### Similar de novo motifs found

|  |  |  |  |  |  |  |  |
| --- | --- | --- | --- | --- | --- | --- | --- |
| Rank | Match Score | Redundant Motif | P-value | log P-value | % of Targets | % of Background | Motif file |
| 1 | 0.761 | A C G T C G T A A C G T A C T G A G T C A G T C C T G A C G T A A C T G A G T C | 1e-28 | -65.293932 | 0.35% | 0.07% | motif file (matrix) |
| 2 | 0.631 | A C T G A C T G A C G T A T G C A T C G A C T G A C T G A G T C A G T C A C G T | 1e-24 | -56.826693 | 0.28% | 0.05% | motif file (matrix) |
